# TEACHING MODEL FOR EVALUATION OF THE ABILITY AND COMPETENCE PROGRESS IN ENDOSUTURE IN SURGICAL SKILL LABORATORY

**DOI:** 10.1590/0102-6720201700040007

**Published:** 2017

**Authors:** Luiz Gonzaga de MOURA-JÚNIOR, Almino RAMOS, Josemberg Marins CAMPOS, Álvaro Antônio FERRAZ, Hermano Ângelo Lima ROCHA, Grijalva Otávio COSTA

**Affiliations:** 1 Laboratory of Surgical Skills, Nucleus of Experimental Surgery, Hospital das Clínicas, Federal University of Pernambuco, Recife, PE, Brazil

**Keywords:** Laparoscopy, Suture techniques, Psycho-motor performance, Minimally invasive surgical procedure, Graduate medical education, Laparoscopia, Técnicas de sutura, Procedimento cirúrgico minimamente invasivo, Desempenho psicomotor, Ensino de graduação em medicina

## Abstract

*****Background*** ****:**

Laparoscopic manual suturing is probably the most difficult skill to be acquired in minimally invasive surgery. However, laparoscopic exercise endo-sutures can be learned with a simulator and are of great practical importance and clinical applicability, absorbing concepts that are immediately transferred to the operating room.

*****Aim*** ****:**

To assess the progression of skills competence in endo-sutures through realistic simulation model of systematized education.

*****Method*** ****:**

Evaluation of the progression of competence of students in three sequential stages of training in realistic simulation, pre-test (V.1), teaching concepts (V.2) and training station for absorption of video concepts in surgery - ergonomics, stereotaxia, ambidexterity, haptic touch, fucral effect, applied in the manufacture of points corresponding to a Nissen fundoplication, in endo-suture for realistic simulation.

*****Results*** ****:**

All students who attended the course absorbed the video concepts in surgery; most participants showed steady and continued improvement and during the stages of training, obtained progression of appropriate skills, defining competence and validation of the teaching model to achieve proficiency.

*****Conclusions*** ****:**

The teaching model was adequate, safe, revealed the profile of the student, the evolutionary powers of the endo-sutures performance and critical analysis of the training to achieve proficiency in bariatric procedures.

## RESUMO

*****Racional*** ****:**

A sutura manual laparoscópica é, provavelmente, a habilidade mais difícil de ser adquirida na cirurgia minimamente invasiva. No entanto, os exercícios laparoscópicos de endossuturas podem ser aprendidos com um simulador e se revestem de grande importância prática, haja vista que tem aplicabilidade clínica, sendo os conceitos absorvidos e transferidos imediatamente para a sala de operação.

*****Objetivo*** ****:**

Avaliar a progressão decompetência de habilidades em endossuturas através de simulação realística em modelo de ensino metodizado**.**

*****Método*** ****:**

Avaliação da progressão de competência dos alunos em três etapas sequenciais de treinamento em simulação realística, pré-teste (V.1), ensinamento dos conceitos (V.2) e estação de treinamento para absorção dos conceitos de videocirurgia - ergonomia, estereotaxia, ambidestria, hapticidade (movimento preciso de instrumentoscom as mão, sem o auxílio ou comando da visão), efeito fucral (movimento invertido de uma balança ou alavanca a partir de um ponto central), aplicados na confecção de pontos correspondentes à fundoplicatura de Nissen em endossutura por simulação realística.

*****Resultados*** ****:**

Todos os alunos que participaram do curso absorveram os conceitos de videocirurgia; a maioria apresentou melhora constante e continuada, e no decorrer das etapas do treinamento,obtiveram progressão de habilidades adequadas, definindo competência e validação do modelo de ensino para atingir a proficiência.

*****Conclusões*** ****:**

O modelo mostrou-se adequado, seguro, revelou o perfil do aluno, a competência evolutiva da performance em endossuturas e análise crítica da carga de treinamento para evoluir até atingir a proficiência nos procedimentos bariátricos.

## INTRODUCTION

According to Leonardi^5^ endoknots, endosutures and endoanastomoses are the most difficult maneuvers in videosurgery. The safe execution of sutures and knots has expanded and enabled the development of more surgeries by this access so more complex procedures are nowadays performed. And, within this context are bariatric operations involving dissection, transections, stapling, gastric and intestinal anastomoses, endosutures for anastomoses, overlapping clamp lines and closures of mesenteric openings to prevent the formation of internal hernias.

Laparoscopic manual suture is probably the most difficult skill to acquire in minimally invasive surgery because of the inherent limitations of videolaparoscopy, among which we can cite: depth perception altered by two-dimensional vision with three-dimensional movements (stereotaxia); dependence on visual-spatial ability and reduced field to work; and triangulation in fixed position of the trocars^7^. On the other hand, laparoscopic exercises in endosuture can be learned with simulator and are of great practical importance, since they have clinical applicability and immediate application in the operating room^2,^ ^6^ ^,^ ^9^ ^,^ ^11^.

The Pernambuco Chapter of the Brazilian Society of Bariatric and Metabolic Surgery, in partnership with the Bariatric and Metabolic Surgery Service of the Department of Surgery of the Federal University of Pernambuco Medical School, Recife, PE, Brazil, systematically conducts lato sensu postgraduation, theoretical and practical, intended for residents and young surgeons who are interested in advancing in bariatric videosurgery. In this way, the focus was to ensure continued education and to effectively and safely extend bariatric procedures with training stations, participation in operations (gastric bypass and vertical gastrectomy) and monitoring with preceptors, until complete autonomy in the execution of the procedure by the student.

In these courses, called BariUp, students attend practical classes in realistic simulation in the Laboratory of Surgical Skills of Hospital of Clinics of the Federal University of Pernambuco, with stages of skills progression and competence in endosutures, aiming to transfer the absorption of psychomotor concepts to the operating room in the technical execution, next to the patient.

Thus, the objective of this paper was to evaluate the progression of competence of endosuture skills through realistic simulation in a methodological teaching model.

## METHOD

This is a prospective, analytical, observational, paired study with 11 students, nine men (82%) and two women (18%), eight Brazilians (73%) and three foreigners (27%), all surgeons of various specialties and different levels of experience in videosurgery, who voluntarily were enrolled in the course and agreed to participate to evaluate the progression of competency in endosuture skills, comparing the evolution between the beginning (pre-test - V.1) and the end of the training (post-test -V.3).

Each student was randomly assigned (drawn and numbered from 1 to 11) to perform the tasks a real abdominal cavity simulator (EndoSuture Training Box® - Figure 1A and 3DMed® - Figure 1B), a needle holder, Ethibond^**®**^ 2.0 suture threads, with a 2.5 cm cylindrical needle and a thermoplastic elastomer (RS^**®**^ ) synthetic suture plate, with self-retaining, parallel edges to perform the surgical stitches.

**FIGUREA 1:**
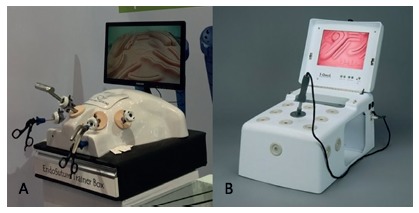
A) EndoSuture Training Box® simulator; B) 3DMed® simulator

The work was divided in three distinct stages: V.1, which consisted in evaluating the level of previous proficiency in videosurgery; V.2, to verify the absorption of the teaching concepts in the methodology of the endosuture model; and V.3, the final profile, after the training, through the execution of endoknots with six stitches and five knots in the time up to 18 min. They did in abdominal cavity simulators a realistic simulation of Nissen fundoplication for hiatal hernia correction, which on average applies two stitches on the diaphragmatic pillar and four on the gastrogastroplication. All the stitches of each student were photographed at the end of the execution, and the execution times noted in the three steps.They were tabulated and classified according to the Scale of Progression of Skills and Proficiency in Video Endosuture^6^.

In the first station (V.1) after the draw of the 11 simulators for the individual performance, the students performed the stitches, according to the previous experience in videosurgery and in endosuture, without any orientation, being delivered to each one, the surgical instruments and the threads for suture. 

**FIGURE 2:**
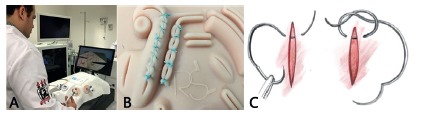
Second station: A) simulator exercises; B) suture device; C) performing C e D letters

In the second station (V.2) students received theoretical classes of the concepts of videosurgery: ergonomics, triangulation, ambidexterity, stereotactic, hapticity (precise movement of instruments with the hands, without aid or command of vision), fucral effect (inverted movement of a scale or lever from a central point), videosurgery films, configuration demonstration, spatial formatting and constructivity of the adjusted knot (Figure 2), management, familiarization of the surgical instruments and the perception of the new work environment in the simulator, notion of depth and reference of intracavitary structures, trocar triangulation and positioning. In addition, the exercises were performed with a suture loop, grip, apprehension, assembly, correct apposition and needle angulation in the tissue penetration, with immediate execution of the task, confection of six stitches and five knots, adjusted, symmetric, according to the proposed methodology.

Then, in the third station (V.3), endosuture exercises were performed with 4 h of training, in two shifts of 2 h each, with an interval of 1 h for rest between steps. All students performed the proposed task (Figure 2A and 2B), with four preceptors’ orientations: loop maneuver with long braided thread, going and returning, with the right and left hands - to enable ambidexterity -, separate sutures, continuous sutures, with the eventual or necessary correction of body movements and spatial positioning, erratic and inadequate maneuvers. At the end of the training steps, they repeated the task consisting in the execution of six stitches with five adjusted knots. This practice was timed and photographed (Figure 2B), for evaluation and comparison of the results in the three stations.

### Statistical analysis

Quantitative categorical results were presented as percentages and counts, numerical results in the form of central trend measures, as well as box-plot type graphs. Normality tests were performed for the numerical variables and the linear regression and Kendall tau B tests were performed to compare variables, as appropriate. Comparisons with p value up to 0.10 were considered significant. The data were tabulated and analyzed by the SPSS software (Statistical Package for the Social Sciences), v23, SPSS, Inc. for the analysis and evaluation of data collected.

## RESULTS

It was observed that the majority of the participants presented constant and continuous improvement during the training stages. Two trainees decreased the pre-test proficiency (V.1) at the execution time of step 2 (V.2), one from good to good and another from good to regular to adapt to the new training model. Then in training, evolved to optimal proficiency (V.3). Of four trainees with insufficient profile, only one remained insufficient (25%), which had no experience in endosuture; started without being able to make any stitches, but progressed and finished the training with two being executed. One trainee evolved to regular (25%) and two to the good level (50%). Most (n=6) finished the training with an optimal concept (55%), performing the task between 12-15 min (Table 1B).

**TABLE 1:** A) Results of all participants (number of stitches and classification); B) number of stitches made in the proposed time

Participant	Stage 1		Stage 2		Stage 3	
1	3	Insufficient	3	Insufficient	5	Regular
2	5	Regular	6	Good	6	Optimum
3	3	Insufficient	5	Regular	6	Good
4	6	Optimum	6	Good	6	Optimum
5	6	Good	6	Good	6	Optimum
6	6	Good	6	Good	6	Optimum
7	3	Insufficient	5	Regular	6	Good
8	3	Insufficient	3	Insufficient	5	Regular
9	6	Good	5	Regular	6	Optimum
10	0	Insufficient	0	Insufficient	2	Insufficient
11	4	Regular	5	Regular	6	Optimum
	p=< 0.001*		p=0.016*		p=0.120*	
Training stage			Number of points made in time			
			Average		Median (Max - Min)	
Stage 1			4.09		4 (6 - 0)	
Stage 2			4.55		5 (6 - 0)	
Stage 3			5.45		6 (6 - 2)	

* Wilcoxon test; p=0,065 (linear regression)

**FIGURE 3:**
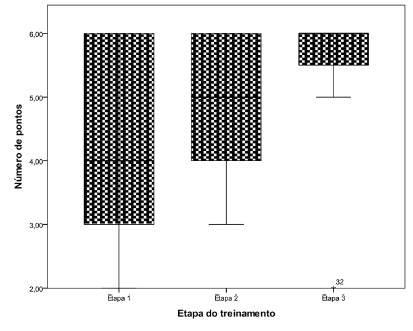
Box graph of the number of stitches made at each stage of the training Bruno traduzir termos em PT

There was an average increase of 1.36 points with the progression of training. In terms of median, each stage was identified improvement of 1 point accomplished. This improvement was statistically significant at the level of 0.1 (Table 1B). Graphically, it can be observed that in addition to the improvement, the students presented a greater constancy in the proficiency level, with much narrower result oscillation bars (Figure 3).

By taking into account the classifications obtained by each participant, it was noticed that in step 1 (V.1) only one student was at the optimal level, while in the final evaluation (V.3) six were able to reach this level. Complementarily, in the first stage, five were insufficient, while in the last one only remained at this level, but progressed in the number of stitches made (from 0 in the first to 2 in the third stage) and absorbed the psychomotor concepts and the methodology. This progression was statistically significant (p=0.01) and this qualification is well perceived by analyzing Tables 1A, 1B, 2 and 3.

**TABLE 2:** Progression of training participants’ proficiency according to the score

	Classification			
Training stage	Insufficient	Regular	Good	Optimum
Stage 1	5	2	3	1
Stage 2	3	4	4	0
Stage 3	1	2	2	6

p=0.009 (Kendall’s tau-b)

**TABLE 3:** Scale of progression of skills in endosutures by videosurger

Scale	Time	V1	V 2	V3	
1.	Insufficient	0 a 3 points	5	3	1
2.	Regular	5 e 4 points	2	4	2
3.	Good	15 -18 min	3	4	2
4.	Great	12 -15 min	1	0	6
5.	Excellent	-< 12min	0	0	0

## DISCUSSION

There was a critical observation of the students at the beginning of the course, when performing the sutures without any didactic guidance and based on their previous experience (V.1), to verify performance and experience in video surgery. When they received orientation from a theoretical-practical model of endorsements (V.2), they followed the orientations in the execution of the tasks, obeying the concepts taught in the course, and they began to adapt to the new spatial perception of constructivity, configuration and formatting of points and surgical stitches. Learning has defined the absorption of psychomotor concepts. The repetition of the movements defined progression of ability, in search of proficiency.

All students improved their performances, evolved at the technical level they were initially in, and self-criticized how much training they lacked in order to have safety and proficiency in performing the procedures in their area. The results showed that the teaching and training model was effective and the students. Besides the improvement, presented constancy in the progression of abilities and in the level of competency in sutures by video surgery.

The Surgical Ability and Proficiency Scale by Videosurgery^8^ consists of the evaluation of the simulated execution of endosutures of a Nissen fundoplication in up to 18 min. The Likert scale (Insufficient - level I, Regular - II, Good - III, Optimum - IV and Excellent - V) was applied to stratify the technical suturing level of the students who participated in the course, defining where they were before the course (V1); in what conditions did they reach the end of training (V3); and the need for additional training load to acquire skills to achieve the excellent proficiency plateau represented by level 5 (Table 2). Although no student was able to achieve the maximum level of proficiency by running the six points with five stitches in less than 12 min, all of them progressed in the ability to execute endosutures. These results demonstrate that, despite the absorption of the concepts of the teaching method, it is necessary to continue training to reach the maximum level. With reduced operative time will benefit the patients in the operating room.

Several studies show the transfer of skills learned in a simulation environment to the operating room, allowing shorter operative time^1^ ^,^ ^10^ ^,^ ^12^ ^,^ ^13^.

According to Domene^3^ the training to act in the robotic access, passes through an equipment of name MIMIC, with programs of simulation with stations of manipulation of objects, movement, use of energy, sutures, etc. The surgeon is aware of his or her performance by assessment that appears immediately after exercise, guiding in what needs improvement, or if it has been performed correctly. The surgeon can thus become familiar with the equipment, and in exhaustive training, perform their initial procedures with more skill and precision, reducing the learning curve (as already demonstrated in controlled studies) and possibly reducing the risks of occurrences of accidents and complications that can occur in this curve, either by the open, laparoscopic or robotic route.

Pupulim et al.^11^ inform that National Academies of Science - BIO 2010 Commission recommends the use of appropriate technology to enhance students’ understanding of the study. Innovations in teaching strategies can result in clear evolution in the application of the taught content and also raise greater interest of the trainee and consequently adherence to the teaching model. In order to achieve excellence, one must first have complete theoretical-practical knowledge about what one wishes to perform, train repeatedly until quality and skill are achieved for the safe surgical act.

The simulator EndoSuture Trainer Box® (Figure 1A), used in the endosuture exercises of the Bari-Up course, was designed and validated by Moura Júnior^8^. It presents several features in teaching the operative technique, and can be seen through an 80” TV monitor where the trainees see the movements of the preceptor, and try to repeat them in simultaneous and continuous act. This facilitates the absorption of psychomotor concepts. It can even be coupled to video surgery equipment or robotic platform, approaching to the maximum of the realistic simulation with the performance of two trainees, one in surgery and the other assisting in the camera or the operative procedure, optimizing the instrument.

The mandates of the Accreditacion Council on Graduate Medical Education state that long-term assessment of the surgical competence of residents is mandatory in the curriculum and the skills laboratory should be an integral component of the surgical residency training program. Hammond, Katchum and Schwuartz^4^ developed models of laboratory practice and perceived the evolution of the participants with the training, indicating evolution of surgical skills facilitated with progressive increase of proficiency among all the participants: trainees and instructors showing high degree of satisfaction, fidelity to the model of teaching, gaining technical experience, and with low stress in these repeated practices.

The Brazilian universities present an absence or deficiency of laboratory of experimental surgery, surgical and clinical skills in undergraduate courses, when, on the other hand, there is a worldwide tendency to equip medical courses with laboratories, administering compulsory practices through the simulation, besides composing elements that favor research and continuing education. The presence of the skill laboratories in school hospitals with medical residency program (general, digestive, urological surgeries, or endoscopy, anesthesiology and clinical specialties, as it happens in the Nucleus of Experimental Surgery, Hospital das Clínicas, Federal University of Pernambuco), is important to better train the participants of the programs.

## CONCLUSIONS

This model of continuing education, including endosuture training, proved to be adequate and safe. It revealed the profile of the student, the evolutionary competence in performing endosutures, and the critical analysis of the laboratory training needed to achieve proficiency in bariatric procedures.
